# ESR statement on portable ultrasound devices

**DOI:** 10.1186/s13244-019-0775-x

**Published:** 2019-09-16

**Authors:** European Society of Radiology (ESR), Dirk-Andre Clevert, Vincent Schwarze, Christiane Nyhsen, Mirko D’Onofrio, Paul Sidhu, Adrian P. Brady

**Affiliations:** Vienna, Austria

**Keywords:** Portable ultrasound devices, Hand-carried ultrasound, Ultrasound diagnostics

## Abstract

The use of portable ultrasound (US) devices has increased in recent years and the market has been flourishing. Portable US devices can be subdivided into three groups: laptop-associated devices, hand-carried US, and handheld US devices. Almost all companies we investigated offer at least one portable US device. Portable US can also be associated with the use of different US techniques such as colour Doppler US and pulse wave (PW)-Doppler. Laptop systems will also be available with contrast-enhanced US and high-end cardiac functionality.

Portable US devices are effective in the hands of experienced examiners. Imaging quality is predictably inferior to so-called high-end devices.

The present paper is focused on portable US devices and clinical applications describing their possible use in different organs and clinical settings, keeping in mind that patient safety must never be compromised. Hence, portable devices must undergo the same decontamination assessment and protocols as the standard equipment, especially smartphones and tablets.

## Key points

- Portable US devices could be indicated for abdominal, cardiac, lung, obstetric, paediatric, vascular, and trauma scanning
- Portable US devices can be used by different healthcare professionals.
- Portable US devices can be associated with the use of different ultrasound techniques such as colour Doppler US and PW-Doppler.
- Portable devices can considerably reduce the overall time required for performing an ultrasound examination at the bedside.
- Portable US devices are effective in the hands of experienced examiners but will not replace a high-resolution US examination.
- Patient safety and high standards of hygiene must be maintained.
- Adequate image and report storage are mandatory.

## Patient summary

Portable and handheld Ultrasound (US) devices are being used increasingly in clinical practice today. Their use is particularly important in situations where time is of the essence (emergency room, intensive care), or the location favours portable devices (remote locations, doctor’s office, etc.). Regardless of the circumstances, adequate user training and competent use of the device are essential. Furthermore, the use of these devices requires protocols for decontamination and data protection – in relation to data collection, transmission, and confidentiality. Overall, the quality of the devices tested and reported on in this report allows responsible clinical use of the devices.

## Introduction

The use of portable ultrasound devices (PUD) has increased in recent years and the market has been flourishing. Formerly only offered in specialised departments as bulky and expensive machines, ultrasound has recently moved to the bedside and become more affordable. At present, PUDs are mainly used by non-radiologist units such as in internal medicine and intensive care units or in pre-hospital settings [1, 2] and allow for complementing clinical examination and providing immediate visual correlates of clinical findings. The idea of an “ultrasound stethoscope”, in addition to taking a history from and clinical examination of patients, is a reality nowadays. Both tools are operator dependent; practice and experience are critical for developing an adequate skill level. In an American study of cardiology practice, first-year medical students achieved the correct diagnosis in 75% of cases by using ultrasound, compared to cardiologists who, by means of clinical examination, arrived at the correct diagnosis in only 49% of the cases [3].

Portable ultrasound devices can be subdivided into three groups: laptop-associated devices, hand-carried (HCU), and handheld (HHUSD) systems. Almost all companies we investigated offer at least one portable ultrasound device.

The big advantages of PUD lie in time saving (booting time, transfer, bedside positioning), e.g., at the bedside or in prehospital situations. On the other hand, drawbacks are the limited battery runtime, the narrowed field of vision, and poor penetration. So far, miniaturised devices may not guarantee adequate image quality [4]. Ongoing research needs to be done to safeguard sufficient resolution in mobile ultrasound devices.

Should portable devices be used, in particular in conjunction with smartphones and tablets, an adequate decontamination assessment is mandatory before first use and strict hygiene protocols must be in place at all times. Patient safety must not be compromised. Image storage should be considered before introducing mobile devices in daily clinical practice. Images and formal reports of all ultrasound studies must be available in the patient records for further reference [5, 6].

## Fields of application

PUDs are mainly used in a small number of clinical specialties and situations. One major field is trauma medicine since ultrasound devices are directly accessible, non-invasive, and inexpensive. The focused assessment with sonography for trauma (FAST) is a crucial component of the trauma care algorithm to assess pericardial or pleural effusions, free intraabdominal blood, and also pneumothoraces. Furthermore, ultrasound helps to identify haemodynamically unstable patients by assessing the status of the inferior vena cava (IVC). As ultrasound devices are getting smaller and portable, in the pre-hospital setting they help the rapid evaluation and triage of victims, e.g. in the context of a mass casualty incident (MCI). A proposed protocol for a comprehensive ultrasound evaluation of MCI victims is the so-called “CAVEAT” exam – chest-abdomen-vena cava or vascular extremity in acute triage [7]. More advanced technologies will guarantee rapid transfer of the point-of-care ultrasound findings to receiving hospitals to provide best medical care.

In several studies, PUD performed efficiently as a tool for screening for abdominal aortic aneurysms in the outpatient setting [8], and proved promising when used by different health care providers (nurses, physical therapists, and physicians) in the assessment of haemarthrosis in haemophiliac patients [9].

A substantial proportion of American rheumatologists routinely use point of care ultrasound (POCUS) to evaluate joint effusions and erosions and abnormalities of the tendons [10].

In addition, PUD proved to be suitable for detecting intrahepatic ductal stones, gallstones, hydronephrosis, and also for volume assessment in patients on haemodialysis or those with acute kidney failure [11].

Besides diagnostic use, portable ultrasound devices were reported to be a feasible guiding tool for interventions, e.g., aspiration/drainage of ascites [12], central venous cannulations, thoracocentesis, or pericardiocentesis [13].

Portable US immediately after MD-CT helps to narrow down the differential diagnosis of hepatic and pleural lesions with minimal additional effort in time and organisation [14].

## Hand-carried ultrasound systems

**Fig. 1:**
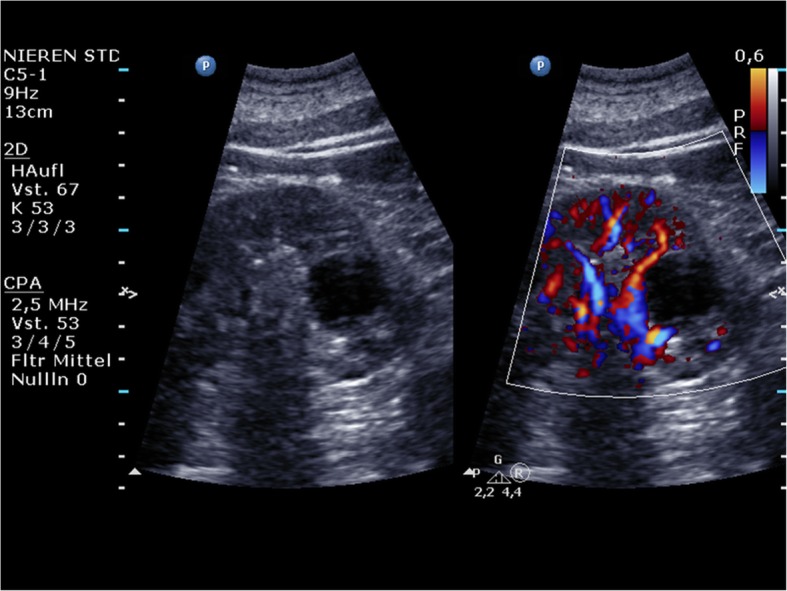
Philips Healthcare CX50: A renal cyst (left) that shows thin walls without any septa, calcifications, solid components, or contrast enhancement. There is no vascularisation of the cyst visible in colour Doppler mode (right)

**Fig. 2:**
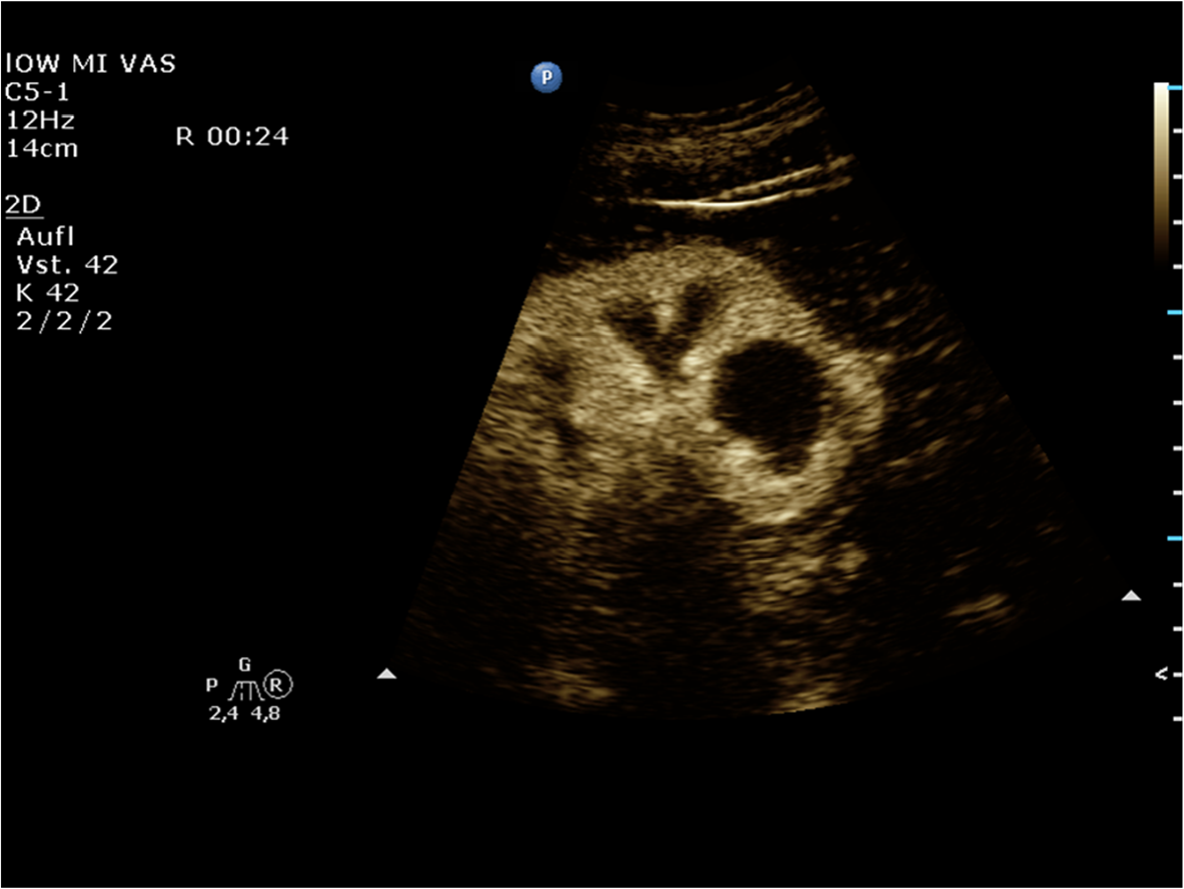
Philips Healthcare CX50: No contrast enhancement of the cyst can be detected; thus, the cyst can be classified as Bosniak type I

Most of the leading ultrasound companies have a hand-carried ultrasound system in their portfolio. Examples include:

- The hand-carried Philips Healthcare CX50 CompactXtreme was released in 2008. It was particularly designed for mobile echocardiography. It can be mounted on a very flexible cart or simply be carried by a handle. The battery run time is about 30 minutes. The CX50 allows for immediate echocardiography on intensive care units, in the emergency room, in the operating theatre as in out-patient clinics (Figs. 1, 2).
- The Sonoace R3 by Samsung can be used for many clinical applications, e.g., for gynaecolgical, abdominal, neonatal, or cardiac issues with curvilinear, linear array, or endocavity curvilinear array probes.

## Handheld ultrasound devices

Handheld ultrasound devices (HHUSD) can be taken anywhere and perfectly fit into a physician’s coat pocket. Many companies produce HHUSDs. Examples include:

- Fujifilm’s SonoSite iViz features a one-handed user interface that connects to a phased, curved, or linear transducer, while Doppler is also available. Stored studies can be exported via USB, DICOM, email, or uploaded to the cloud.
- Clarius offers wireless HHUSDs that run with iOS and Android. Clarius Clip-Ons allow the user to have a multi-purpose scanner in one device.

The following sections describe the features of specific devices, as examples of succeeding generations of HHUSDs. They are not intended to imply that the described devices are the only, or the best example in each category, but have been chosen simply as illustrative examples.

## The first generation of HHUSD: Acuson P10 Siemens

**Fig. 3:**
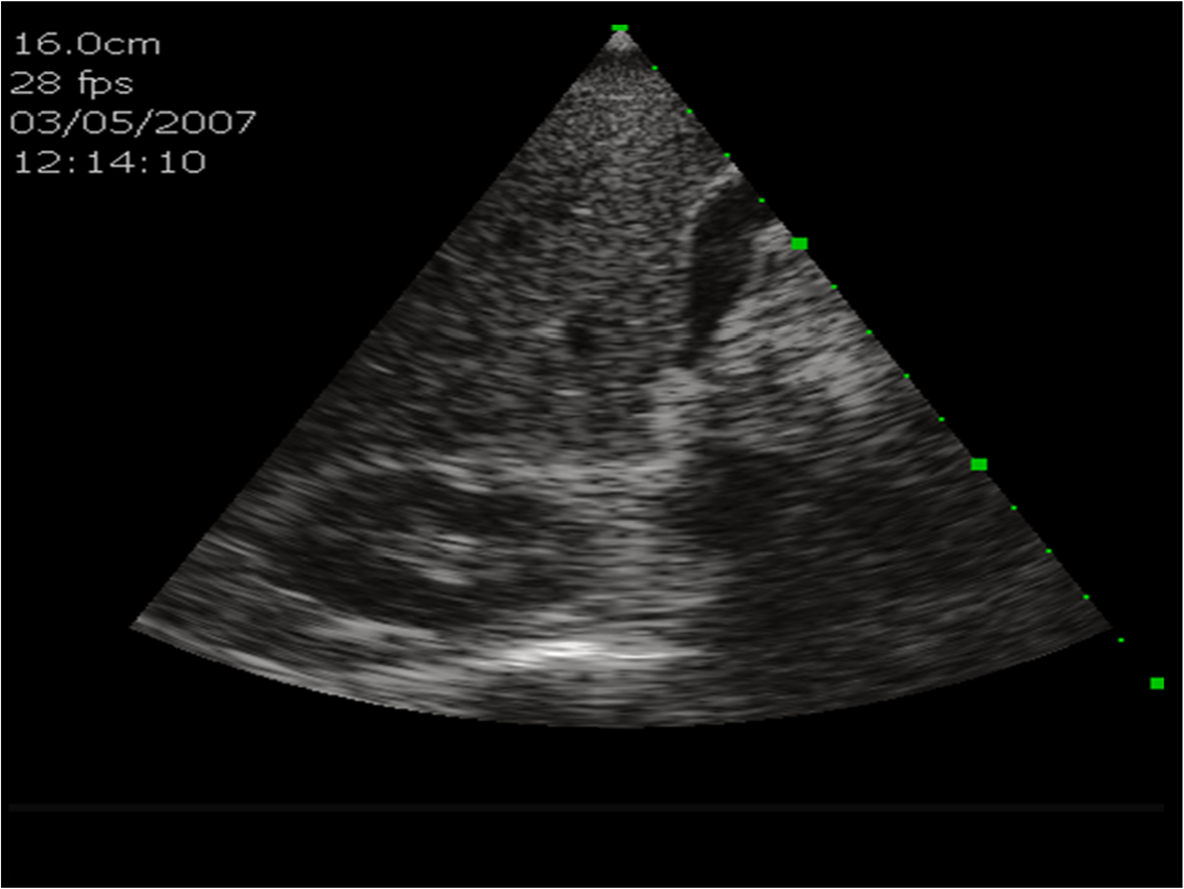
Acuson P10 Siemens: B-scan ultrasound of the liver, including the gall bladder and the right kidney

The Acuson P10 system by Siemens Medical Solutions was introduced in 2007 as the first portable ultrasound device. It weighs about 0.730 kg and was designed for immediate and easy use in emergency medicine, cardiology, and obstetrics with an intuitive Personal Digital Assistant (PDA) interface. It features a 3.7-inch touch-screen LCD display with 640 x 480 pixels with a wide viewing angle and a lithium battery which provides a run time of approximately 60 minutes. According to the manufacturer’s manual, the start-up time is 10 seconds. The Acuson P4-2 phased array transducer allows abdominal, renal, obstetric, transthoracic, and cardiac applications in the context of emergency medicine. The frequency range is 2–4 Mhz and allows for 2–24 cm of display depth with up to 28 frames per second. Examinations can be stored on an SD memory card up to 2 GB or transferred via USB. The Acuson P10 offers 2D-mode imaging in fundamental and harmonic modes (Fig. 3). Besides abdominal diagnostic use, P10 ultrasound devices were used in cardiac imaging [4, 15–18].

## The second generation of HHUSD: GE Healthcare VScan with Dual Probe

**Fig. 4:**
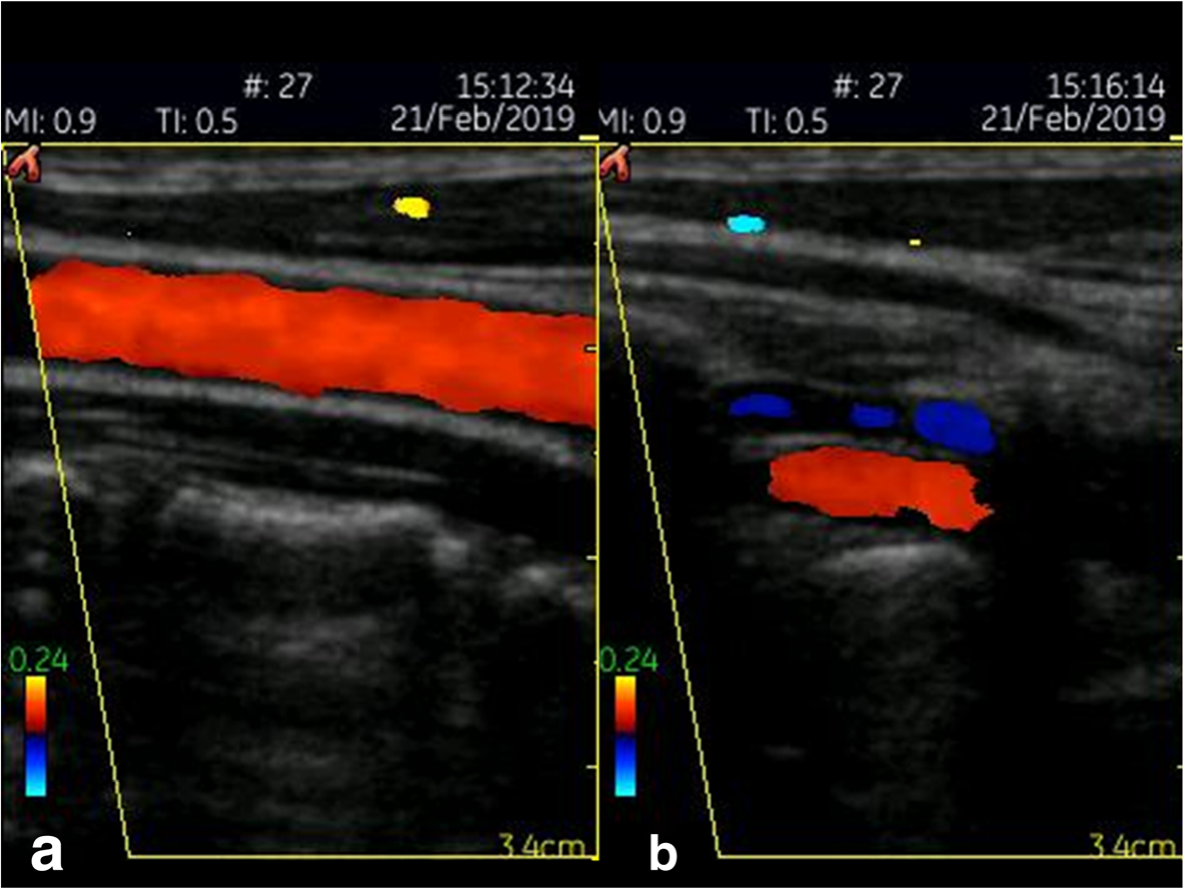
GE Healthcare VScan (linear array transducer): Colour Doppler image of the carotid (**a**) and vertebral artery (**b**)

**Fig. 5:**
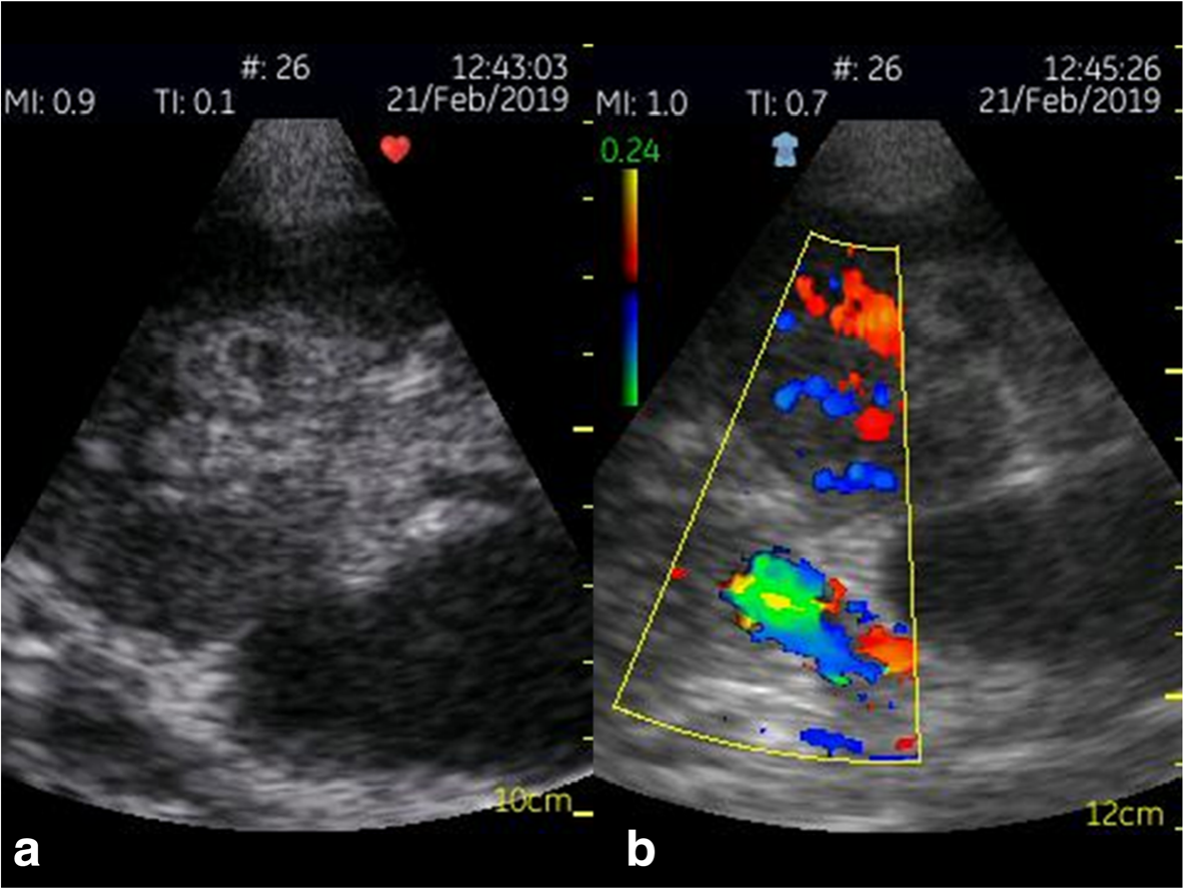
GE Healthcare VScan (phased array transducer): B-scan ultrasound (**a**) and colour Doppler (**b**) of a transplant kidney

The recent GE Healthcare VScan with Dual Probe is the first kind of ultrasound devices that features two transducers in a single probe. The VScan with Dual Probe is designed for a broad spectrum of clinical applications, including ultrasound-guided catheter placements, cardiac, abdominal, thoracic, and foetal issues. It weighs 0.436 kg and has a 3.5-inch display allowing for 240 x 320 pixels resolution. The phased array transducer allows for a 75-degree field with a maximum depth of 24 cm for B-mode and for colour Doppler mode with an angle of up to 40 degrees. The frequency range is 1.7–3.8 MHz. The linear array transducer allows for a maximum depth of 8 cm for B-mode and for colour Doppler mode. The frequency range for the linear array transducer is 3.4–8.0 MHz. Data can be stored on microSD (HC) cards or transferred via USB. According to the manufacturer’s manual, the battery runtime is about 60 minutes. Besides abdominal diagnostic use, Vscan ultrasound devices were used in MSK, cardiac imaging, OB/GYN, and emergency settings [13, 19–25] (Figs. 4, 5).

## The third generation of HHUSD: Philips Lumify

The Philips ultrasound Lumify works with a compatible smart device (e.g., smartphone or tablet). Using the Lumify, three different transducers are available: an S4-1 broadband sector array (4 to 1 MHz), a C5-2 broadband curved array transducer (5 to 2 MHz) and an L12-4 broadband linear array transducer (12 to 4 MHz). The system allows 2D, steerable colour Doppler, M-mode, advanced XRES, and multivariate harmonic imaging and SonoCT. By using different transducers, applications can be extended to include Cardiac, OB/GYN, Lung, Abdomen, FAST, Soft Tissue, Vascular, Superficial, and Musculoskeletal. For the first time, a compatible smartphone or mobile ultrasound device can be used to plug in an ultrasound transducer. All necessary beamforming and data management is done in the probe. The mobile smart device is only necessary for the battery supply and the display. The Lumify app must be uploaded on the mobile device to enhance imaging. Advanced imaging algorithms are automatically available and create the image. By using the touch screen of the mobile device, depth, gain, power, and colour can be optimised.

**Fig. 6:**
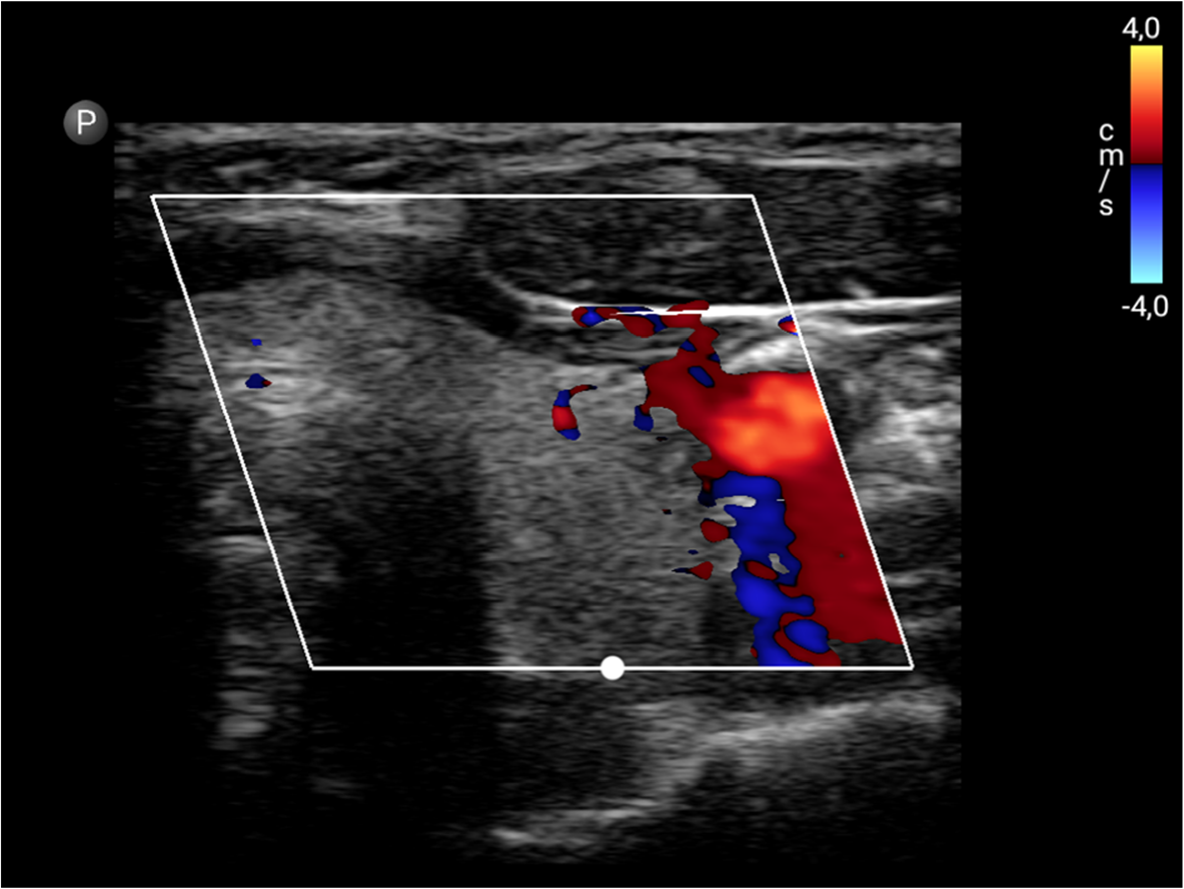
Philips Lumify linear transducer: Colour Doppler of the left thyroid lobe including the carotid artery

**Fig. 7:**
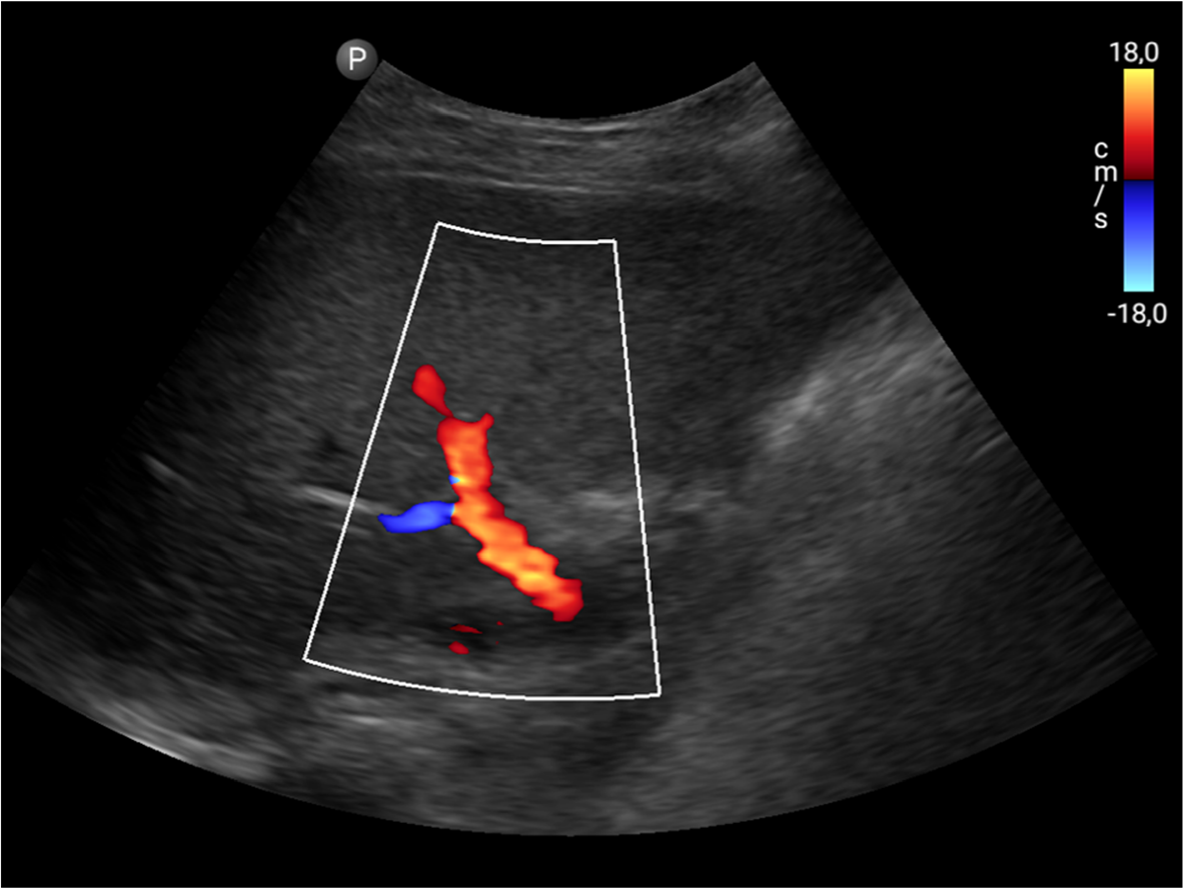
Philips Lumify curved array transducer: Colour Doppler imaging of the portal vein

In a study by Miller et al, 56 patients scheduled for either reconstructive or aesthetic surgery were evaluated preoperatively and/or intraoperatively by a single surgeon with a linear 12–4 probe using the Philips Lumify device. For patients undergoing flap reconstruction, potential donor sites were imaged in order to locate the largest perforator. For patients undergoing abdominal procedures, intraoperative visualisation of the abdominal muscular layers was used for the delivery of anaesthesia during transversus abdominis plane block. Lastly, the superficial fascial system was subjectively evaluated in all preoperative patients. The conclusion of the study was that the newest, miniaturised colour Doppler ultrasound technology has a variety of applications that may improve patient outcomes and experience in plastic surgery [26]. Besides abdominal diagnostic use, the Lumify and the Visiq ultrasound devices were used in abdominal imaging, emergency, and general imaging [26–29] (Figs. 6, 7).

## Outlook: Butterfly’s iQ

**Fig. 8:**
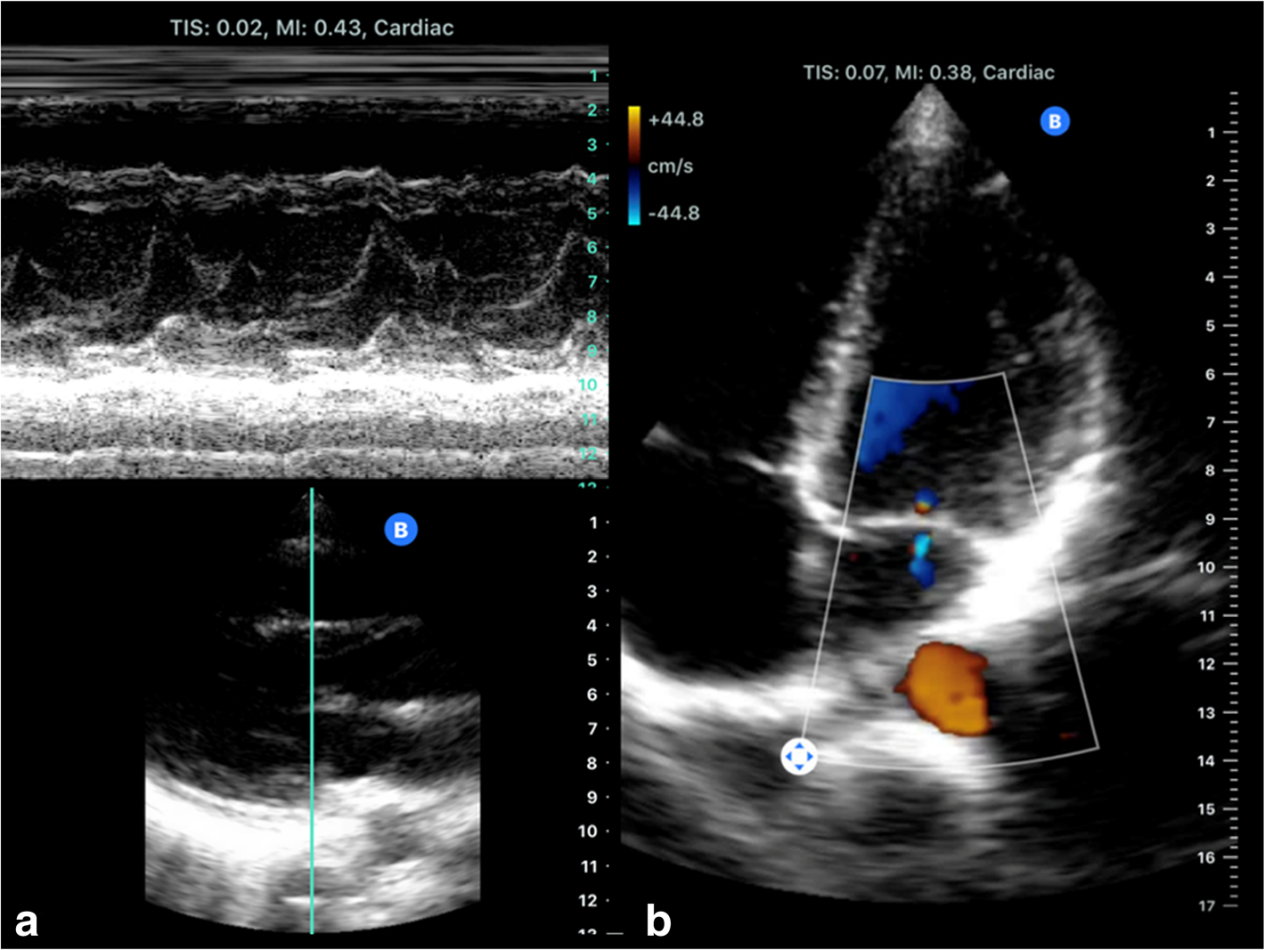
Butterfly’s iQ emulate phased transducers: Cardiac imaging including M-Mode (**a**) and four chamber view with colour Doppler (**b**)

**Fig. 9:**
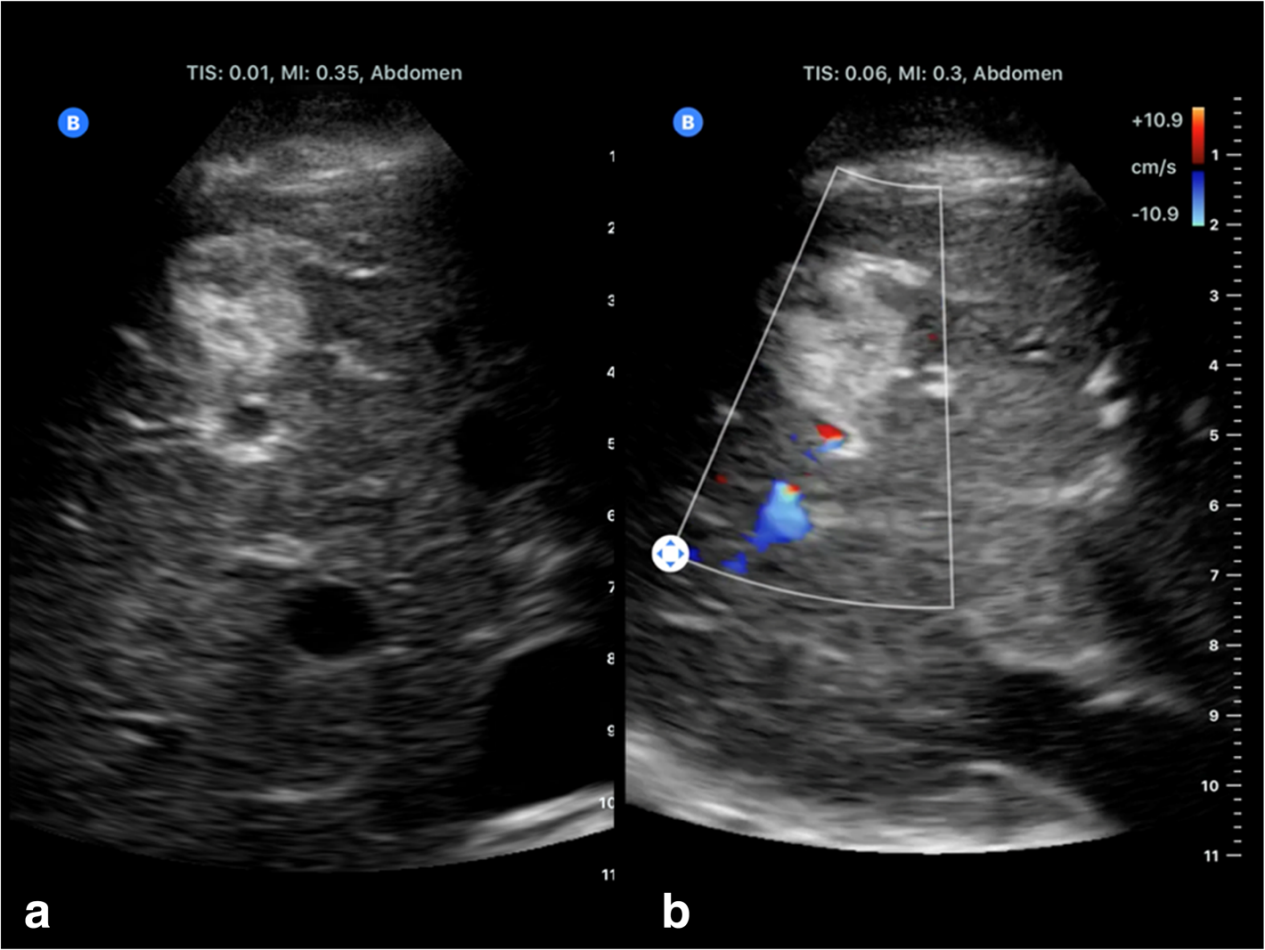
Butterfly’s iQ emulate curved transducers: B-scan ultrasound (**a**) and colour Doppler (**b**) of a liver haemangioma

As a very recent innovative start-up, the Butterfly Network's handheld ultrasound device features a silicon chip (2D array, 9000 micro-machined sensors) instead of piezoelectric crystals to induce ultrasound waves (“Ultrasound-on-Chip technology”). This allows it to emulate curved, linear, or phased transducers at any time in M-, B-mode or colour Doppler with 2–30 cm scan depth. It weighs only 0.313 kg and is connected to a smartphone. The battery run time is 120 minutes and the wireless full recharge takes up to 5 hours. Moreover, the ultrasound findings can be uploaded to the Butterfly Cloud, so any expert with access can help evaluate the sonographic findings. By using artificial intelligence algorithms, the position of the probe can be adjusted to meet the requirements of the user (Figs. 8, 9).

Healthcare workers or paramedics might be equipped with handheld ultrasound devices like Butterfly’s iQ and artificial guidance with the immediate ultrasound correlates with rapidly recognising serious health issues. In the future, even patients might be provided with handheld ultrasound devices, so their caring physicians might, without directly seeing the patient, evaluate uploaded ultrasound findings. Furthermore, healthcare systems in developing countries may benefit immensely from affordable ultrasound devices [30, 31].

Hand-held devices open up new possibilities of imaging at the point of care, in whichever setting this is needed. However, adequate training of ultrasound users, image and report storage for further reference, and high standards of hygiene are mandatory. Patient safety must not be compromised.

## Conclusion

The newest, miniaturised handheld ultrasound devices technology has a variety of applications that may improve patient outcomes and experience [32]. The overall time required for performing an ultrasound examination at the bedside can be considerably reduced if a portable device is used instead of a mobile system [33]. High standards of hygiene must be maintained. Images and reports must be stored in patient records for further reference.

## Data Availability

Not applicable.
